# A Case of Concomitant Plasmodium falciparum Malaria and Bacillus cereus Bacteremia in a Returning Traveler From Tanzania

**DOI:** 10.7759/cureus.32969

**Published:** 2022-12-26

**Authors:** Marcos Garcia, Joya-Rita Hindy, Antoine Boustany, K V Gopalakrishna

**Affiliations:** 1 Internal Medicine, Cleveland Clinic Fairview Hospital, Cleveland, USA

**Keywords:** diarrhea, plasmodium falciparum, bacteremia, malaria, bacillus cereus

## Abstract

Malaria has been associated with bacterial co-infections, but the importance of bacterial co-infections in uncomplicated malaria is poorly described. We report a unique case of a 27-year-old female with concomitant Plasmodium falciparum and Bacillus cereus bacteremia who acquired those infections while traveling in Tanzania but became ill only after returning to the United States. Blood parasites screen revealed Plasmodium falciparum and blood cultures obtained at presentation showed Bacillus cereus. Even after completing treatment for malaria, she continued to have abdominal pain and watery diarrhea, which improved only after IV vancomycin. Bacillus cereus bacteremia cases are reported in travelers and immigrants returning from countries where malaria transmission occurs, mainly from sub-Saharan Africa but co-infection with Plasmodium falciparum and Bacillus cereus has not been described in the literature yet. In this case, malaria symptoms resolved after targeted treatment was initiated but persistent diarrhea improved only after appropriate therapy against Bacillus cereus. Persistent watery diarrhea and dehydration in patients with malaria should raise concerns about Bacillus cereus co-infection.

## Introduction

Malaria has been associated with bacterial co-infections and in particular with bacteremia [1]. The most commonly isolated bacteria from the blood of malaria patients are non-typhoid Salmonella species and other Gram-negative bacteria [2]. In uncomplicated malaria, the significance of bacterial co-infections is poorly understood, and the symptoms of malaria and bacterial infections may be similar.

In individuals with malaria, intestinal translocation of bacteria and increased erythrophagocytosis have been suggested as possible mechanisms for increased susceptibility to bacteremia [3]. The malaria-induced hemolysis and the production of proinflammatory cytokines by dendritic cells are proposed mechanisms that may negatively impact the ability of phagocytic cells to respond to bacteremia, thus facilitating the spread of bacteria [4].

Due to limited awareness in non-endemic regions, imported malaria in high-income settings can have high mortality and risk of delayed diagnosis [5]. We report a unique case of concomitant Plasmodium falciparum and Bacillus cereus bacteremia in a previously healthy young woman. She acquired the infection while traveling in Tanzania but became ill after returning to Ohio.

## Case presentation

A 27-year-old female presented to the emergency department (ED) in Ohio because of fever, nausea, vomiting and watery diarrhea for three days. Thirteen days before ED presentation she had returned from a 30-day trip to Tanzania. While in Tanzania, she was asymptomatic and stayed in an urban environment. On the day of her trip back to the US, she developed watery diarrhea and nausea. Three days later, she complained of fevers that were occurring multiple times per day, worsening diarrhea, and dehydration. She then sought medical care. She denied having any headache, skin rash, respiratory symptoms or sore throat. While in Tanzania, she reported mosquito bites. She endorsed eating home-cooked meals and drinking bottled water, but she drank tap water a few times while in Tanzania. She denied any sick contact. She was advised to take a prescription medication for malaria prevention, but could not get it before traveling.

The patient had no relevant past medical history and was not taking medications. She had received vaccines against yellow fever, tetanus, influenza, COVID-19, hepatitis A and meningococcus. She was born and raised in Tanzania and moved to the US when she was a teenager and mentioned no other recent international travel. This trip was her first trip to Africa since she moved to the US.

In the ED, her temperature was 100.9° F, pulse was regular and 120/minute, blood pressure was 149/75, respiration rate was 17/minute and unlabored, and O2 saturation was 99% while breathing ambient air. On physical examination, she appeared uncomfortable. She did not have any jaundice or neck lymphadenopathy. On auscultation, lung auscultation and heart sounds were normal. Her abdomen was tender in the epigastrium, and there was no hepatosplenomegaly. Her neurologic exam was unremarkable.

Laboratory test revealed thrombocytopenia (129 k/uL), her hemoglobin was 12.5 g/dL with signs of hemolysis with a lactate dehydrogenase of 314 U/L. Her aspartate aminotransferase (AST) was 31 U/L and alanine aminotransferase (ALT) was 54 U/L. Her sodium level was 132 mEq/L, potassium 3.1 mEq/L, blood urea nitrogen (BUN) was 12 and creatinine 0.76 mg/dL (estimated glomerular filtration rate 110 ml/min). Initial lactate was 2.4 mmol/L and improved to normal after fluid resuscitation. 

A blood parasite screen was done with a Binax NOW Enzyme Immunoassay (EIA) for Plasmodium spp. and Giemsa stained thick and thin smears were obtained. The tests were confirmatory for P. falciparum with a parasitemia level of 1.9%. Enteric Bacterial Panel by polymerase chain reaction (PCR) of stool specimen was negative for Salmonella, Shiga-like toxin-producing E. coli, Campylobacter, Shigella, enteropathogenic E. coli, enterotoxigenic E. coli, and enteroinvasive E. coli. Ova and parasite examination were negative for amoeba, helminths, or protozoa. Clostridium difficile PCR was negative. On day two of hospital stay blood cultures showed Gram-positive bacilli, confirmed on the next day as a Bacillus cereus species (MALDI-TOF mass spectrometry). Further testing for syphilis, HIV, dengue, Chikungunya and Zika virus were negative.

She was treated for uncomplicated malaria with artemether/lumefantrine for three days. Fever resolved by day three at the hospital but diarrhea and dehydration persisted. Repeat blood smear on day three showed parasitemia < 0.1%. Regarding the B. cereus bacteremia, a decision was made to consider this a pathogen and not a contaminant due to the persistent abdominal tenderness and watery diarrhea requiring IV fluid resuscitation daily. After completing the treatment for malaria on day three, she was started on IV vancomycin 1g every 12 hours on the same day. On the second day of vancomycin her diarrhea improved and on day three of vancomycin diarrhea was resolved. She was discharged on the fifth day of hospital stay, with instructions to take levofloxacin 750 mg daily for additional seven days.

## Discussion

Bacillus cereus is a facultatively anaerobic, spore-forming Gram-positive bacilli. The ingestion of food contaminated with enterotoxigenic B. cereus or the emetic toxin causes infection [6]. The diarrheal-type illness includes profuse watery diarrhea, abdominal pain, and cramping, and sometimes nausea and vomiting [7]. Symptom onset is generally within six to 15 hours of eating food left at room temperature for over two hours. Symptoms normally resolve within 24 hours after onset.

There is limited information available on the incubation period for Bacillus cereus bacteremia in the literature. According to a review article on Bacillus cereus infections, the incubation period for B. cereus bacteremia can range from a few hours to several days, with a median incubation period of 18-24 hours [6]. However, it is important to note that the incubation period may vary depending on the specific strain of B. cereus, the route of infection, and the individual's immune status. One possibility is that the patient's immune system was already compromised due to the presence of malaria, which may have made her more susceptible to developing bacteremia. The observed incubation period for B. cereus bacteremia in the present case was significantly longer than the typical range reported in the literature. Further research is needed to understand the factors influencing incubation periods in this disease [8].

B. cereus bacteremia is rare and reported in the setting of intravenous drug use, central venous lines or mucosal injuries, especially in patients with immunosuppression. The clinical presentation in B. cereus bacteremia can be dramatic, starting with gastrointestinal symptoms and progressing to alteration of consciousness or even infective endocarditis. A 2007 study testing susceptibility of Bacillus species to several antibiotics reported universal resistance to trimethoprim/sulfamethoxazole and beta-lactams [9]. In that study, isolates were susceptible to quinolones, linezolid, streptomycin, tetracycline, tigecycline, and vancomycin based using Sensititre® automated microbroth dilution and Etest® agar gradient diffusion methods [9].

About 2,000 cases of malaria are diagnosed in the US annually, commonly in travelers and immigrants returning from countries where malaria transmission occurs [10]. Co-infection with P. falciparum and B. cereus has not been reported in the literature yet. Here, initially, the positive blood culture was considered possible contaminant, however, the patient was on her third day of malaria treatment and fever resolved on the second day but watery diarrhea persisted. The workup for acute diarrheal illnesses was negative and the patient was more dehydrated, requiring intravenous fluids. After starting vancomycin, the diarrhea improved rapidly and the patient was discharged home.

It is unclear why bacteremia is more common in patients with malaria. Theories have been suggested to investigate the impact of malaria on the immune system. The infection alters the production of pro-inflammatory cytokines by dendritic cells and suppresses the oxidative burst capacity of neutrophils, allowing for sustained bacteria replication [11]. Differently from most bacteria, this phenomenon is well described only for Salmonella coinfection. So, we report a unique case of concomitant P. falciparum and Bacillus cereus bacteremia in a traveler returning to the US from Tanzania. Further studies are required to understand the mechanisms of this co-infection.

## Conclusions

This case report highlights the importance of considering Bacillus cereus bacteremia co-infection in patients with malaria, particularly in those who present with persistent watery diarrhea and dehydration. A thorough medical history and laboratory workup, including blood cultures and parasitemia levels, can aid in the accurate diagnosis of such cases. It is crucial to routinely obtain these tests from febrile patients returning from malaria-endemic areas, as bacterial co-infections may be contributing to unresolved symptoms. This report contributes to a greater understanding of the relationship between malaria and Bacillus cereus bacteremia, and underscores the need for increased awareness and vigilance in the diagnosis and management of these infections.
